# The metabolic adaptation mechanism of metastatic organotropism

**DOI:** 10.1186/s40164-021-00223-4

**Published:** 2021-04-29

**Authors:** Chao Wang, Daya Luo

**Affiliations:** 1 School of Basic Medical Sciences, Nanchang University, Nanchang, 330006 China; 2 Department of Biochemistry and Molecular Biology, School of Basic Medical Sciences, Nanchang University, Nanchang, 330006 China

**Keywords:** Cancer metastasis, Cancer metabolism, Organotropism, “Seed and soil” hypothesis

## Abstract

Metastasis is a complex multistep cascade of cancer cell extravasation and invasion, in which metabolism plays an important role. Recently, a metabolic adaptation mechanism of cancer metastasis has been proposed as an emerging model of the interaction between cancer cells and the host microenvironment, revealing a deep and extensive relationship between cancer metabolism and cancer metastasis. However, research on how the host microenvironment affects cancer metabolism is mostly limited to the impact of the local tumour microenvironment at the primary site. There are few studies on how differences between the primary and secondary microenvironments promote metabolic changes during cancer progression or how secondary microenvironments affect cancer cell metastasis preference. Hence, we discuss how cancer cells adapt to and colonize in the metabolic microenvironments of different metastatic sites to establish a metastatic organotropism phenotype. The mechanism is expected to accelerate the research of cancer metabolism in the secondary microenvironment, and provides theoretical support for the generation of innovative therapeutic targets for clinical metastatic diseases.

## Background

### Cancer metastasis

Metastasis refers to the process by which cancer cells leave the site of cancer occurrence, arrive at distant organs by various ways, and thrive in distant organs, which is one of the hallmarks of cancer cells [1]. Clinically, approximately 90% of cancer patients die from metastatic disease, yet the molecular mechanisms underlying metastasis are poorly understood [2]. It is currently believed that cancer metastasis is a dynamic and complex multistep process known as the “metastatic cascade”. This cascade includes local invasion of surrounding tissues, intravasation to the blood vessel wall, survival in the circulatory system and formation of circulating tumour cells (CTCs), arrest in a distant organ, extravasation as disseminated tumour cells (DTCs), formation of micrometastatic clones and colonization in distant organs to form clinically detectable macrometastatic clones [2, 3].

In 1889, the English surgeon *Stephen Paget* discovered that the organs of cancer metastasis are not randomly but selectively distributed; therefore, he proposed the “seed and soil” hypothesis in which cancer cells are referred to as seeds and the destination of metastasis as represented by soil. Cancer cells can spread similar to seeds but can thrive only in congenial soil [4]. Now, this phenomenon of non-random metastasis is known as “metastatic organotropism”, or “organ-specific metastasis”. The common metastatic organs vary by cancer type and subtype. Prostate cancer often metastasizes to bone [5], while pancreatic cancer and uveal melanoma often metastasize to the liver [6, 7]. Breast cancer often metastasizes to multiple organs, such as bone, lung, liver, brain, and distant lymph nodes, but different subtypes of breast cancer have different propensities for metastatic sites [8–10]. In addition, cancer cells can also exhibit an ordered and hierarchical organ-specific metastatic pattern. For example, colorectal cancer usually first metastasizes to the liver and then to the lung [11]. There are many factors that determine metastatic organotropism, including circulatory system patterns, internal characteristics of the cancer, the tissue- or organ-specific microenvironment, and the interaction between cancer cells and the host microenvironment [3, 4, 12, 13]. Recently, the metabolic adaptation mechanism of cancer metastasis has been proposed as an emerging model of the interaction between cancer cells and the host microenvironment [14], revealing a deeper internal connection between cancer metabolism and metastasis.

### Cancer metabolism

Metabolic reprogramming is one of the important features in the development and progression of cancer, and it is crucial for the survival and growth of cancer cells [15]. A century ago, German biochemist *Otto Warburg* first observed that cancer cells undergo metabolic changes. In the case of sufficient oxygen, the anaerobic oxidation of cancer cells is enhanced, oxidative phosphorylation (OXPHOS) is inhibited, and a large amount of glucose produces lactate through glycolysis in a process known as the “Warburg effect” or “aerobic glycolysis” [16]. With the continued in-depth research, more metabolic characteristics of cancer cells have been gradually revealed [17], and cancer metabolism shows incredible flexibility and plasticity. Ketone bodies are mainly produced by the liver but cannot be consumed by normal adult hepatocytes [18]. However, in the absence of nutrients, hepatocellular cancer cells can induce the expression of OXCT1, a key enzyme in ketone catabolism, by activating the mTORC2-Akt-SP1 signalling axis, thereby utilizing ketone bodies to generate energy and support cancer progression [19]. Another study also found there are alternative fatty acid desaturation pathways activated in liver and lung cancer cells [20]. The urea cycle is the main pathway by which ammonia, a metabolic waste, is eliminated from the body. On the one hand, urea cycle initiators and intermediates can be transferred to other metabolic pathways for anabolism [21]; on the other hand, they can also activate metabolism-sensing signalling pathways and exert biological effects distinct from the classical metabolic pathways, demonstrating the strong waste-recycling capacity of cancer cells [22]. In breast cancer, ammonia can be recycled as a nitrogen source by glutamate dehydrogenase and then used in the synthesis of other amino acids, such as proline and aspartate, to support the growing breast cancer biomass [23]. Normally, ammonia-derived CP in mitochondria cannot enter the cytoplasm for pyrimidine synthesis. However, in non-small cell lung cancer (NSCLC) with KRAS mutation and LKB1 deletion, the expression of carbamoyl phosphate synthase (CPS) 1 increases; this change leads to an increase in the CP, which moves from mitochondria to the cytoplasm. Subsequently, the ammonia-derived CP enters the pyrimidine synthesis pathway as a CPS2 substrate to realize waste recycling and support rapid cancer DNA synthesis and fast cell proliferation [24]. One study discovered "urea cycle disorders" in a wide range of cancers. These disorders involve the transfer of nitrogen from the urea cycle; the activation of the multifunctional enzyme CAD protein composed of CPS2, aspartate carbamoyl transferase and dihydroorotase; and the increase in pyrimidine synthesis [25]. In addition, a recent study found that p53 can transcriptionally inhibit the metabolic enzymes CPS1, ornithine transcarbamoylase and arginase 1 in the urea cycle, thereby inhibiting urea synthesis and ammonia clearance. Accumulation of ammonia can significantly downregulate the mRNA translation of the rate-limiting enzyme ornithine decarboxylase in polyamine biosynthesis, thus inhibiting polyamine biosynthesis and cell proliferation [22]. In summary, cancer cells have extremely high metabolic flexibility and can generate energy, support the integrity of the cell membrane structure, synthesize biological macromolecules and regulate gene expression. These biological processes promote the occurrence, survival and development of cancer.

Similar to metastasis, cancer metabolism is not a static and uniform process but a dynamic and heterogeneous process that is mainly determined by the inherent characteristics of cancer cells and the external environment [15, 26, 27]. The inherent characteristics of cancer cells mainly include oncogenic mutations and the tissue of origin. Oncogene and tumour suppressor gene mutations can regulate the metabolic phenotype of cancer through oncogenic signalling pathways. The PI3K-AKT signalling pathway undergoes recurring mutations in a variety of cancers, which enables cells to take up an increased number of nutrients and synthesize biological macromolecules, even in cases of low levels of extracellular growth factor [28]. The inactivating mutations of oncogenes RAS and MYC can also enhance the uptake of glucose and glutamine, increase the biosynthesis of biological macromolecules and maintain energy and redox balance through different mechanisms [29–31]. P53 is a common tumour suppressor gene that is mutated or deleted in approximately 50% of human cancers. P53 can inhibit glycolysis, enhance OXPHOS and resist oxidative stress [32]. Mutations in metabolic enzymes can also directly regulate metabolic phenotypes. Multiple metabolic enzymes that catalyse the TCA cycle can be mutated; for example, IDH1 and IDH2 mutations can lead to the accumulation of D-2 hydroxyglutarate [33, 34]. SDH and FH mutations lead to the accumulation of succinate and fumarate, respectively [35]. D-2 hydroxyglutarate, succinate and fumarate can affect epigenetic modifications and HIF-1α degradation and participate in the development of cancer [17].

The tissue of origin also affects the metabolic characteristics of the cancer. Through pan-cancer analysis of metabolic gene expression profiles, the metabolic gene expression programme of primary cancer was found to be very similar to that in the normal corresponding source tissues [36, 37]. Moreover, tumours from different tissue origins driven by the same oncogene may have different metabolic patterns. For example, although both are MYC-induced mouse tumours, MYC-induced liver tumours show reduced glutamine anabolism, but MYC-induced lung tumours show increased glutamine anabolism [38]. Kras activation and Trp53 deletion in mouse NSCLC and pancreatic cancer show different types of branched chain amino acid metabolism [39].

**Fig. 1:**
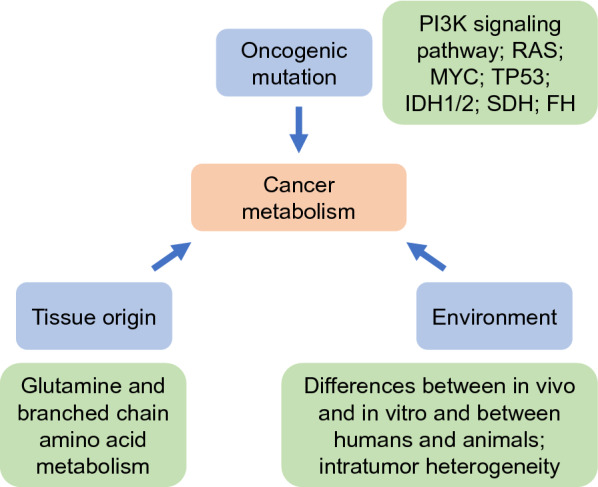
Determinants of cancer metabolism. The inherent characteristics of cancer cells and the external environment together determine cancer metabolism. The inherent characteristics of cancer cells mainly include oncogenic mutations and tissue origin. However, the effect of the environment on cancer metabolism can be greater than that of the intrinsic characteristics of the cancer cells

In addition to the inherent characteristics of cancer cells, the external environment also has an important impact on metabolism, and cancer metabolism exhibits an environmental dependence (Fig. 1). First, differences between the in vivo and in vitro environments lead to differences in cancer metabolism. Ras-driven NSCLC mainly relies on glucose to supply the TCA cycle in the body and mainly relies on glutamine to supply the TCA cycle in medium [40]. Second, environmental differences between animals and humans also lead to differences in cancer metabolism. Uric acid can directly inhibit uridine monophosphate synthase (UMPS) and reduce the sensitivity of cancer cells to the chemotherapy drug 5-fluorouracil [41]; however, the concentration of uric acid in mouse plasma is one-tenth that of human plasma, and therefore the mouse plasma environment shows little effect on UMPS activity or 5-fluorouracil-induced resistance of cancer cells [41]. Subsequently, cells of the same primary cancer located in different environments show metabolic differences. As analysed by preoperative multimodality imaging combined with intraoperative ^13^C-glucose infusions, different areas of NSCLC perfusion showed metabolic heterogeneity [42]. Finally, different cancers in the same environment have similar metabolic patterns. Both gliomas and brain metastases can be fuelled by oxidized glucose and acetate without glutamine oxidation [43]. Therefore, the effect of the environment on cancer metabolism can have an even greater effect than the intrinsic characteristics of the cancer cells.

However, research on how the host microenvironment affects cancer metabolism is mostly limited to the impact of the local tumour microenvironment at the primary site. Few studies have explored how the difference between the primary and secondary microenvironments promotes metabolic changes during cancer progression or how different secondary microenvironments shape different cancer metabolic patterns. Hence, we discuss how cancer cells adapt to and settle in the metabolic microenvironment of different metastatic sites to establish a metastatic organotropism phenotype. The results are expected to accelerate the research of cancer metabolism in the secondary microenvironment and promote the creation of new therapeutic targets for clinical metastatic diseases.

### Metabolic adaptation mechanism of metastatic organotropism

The metabolic adaptation of metastatic organotropism refers to the fact that metabolic pattern of metastatic cancer cells needs to be compatible with the metabolic microenvironment of distant organs to support the survival and growth of metastasizing cancer cells. The metabolic microenvironment that drives cancer metastases to distant organs needs to adapt to acquire sufficient energy, nutrient sources, organ-specific metabolites, and the proper pH, adjust to hypoxic or normoxic conditions, and support metabolic interactions between organ-specific cells and cancer cells. Only a small proportion of DTCs can colonize at a distant site, indicating that the survival and growth of DTCs during colonization requires overcoming significant obstacles. Organ-specific metabolic adaptation can help metastatic cancer cells overcome the obstacles faced when colonizing distant organs and establishing an organotropism phenotype [14].

### Bone

Bone has a high mineral content, extreme hardness, high extracellular calcium concentration, a hypoxic microenvironment and acidic pH [44, 45]. The inorganic phase of bone is mainly composed of mineral hydroxyapatite (HA) crystals, while the bone matrix is rich in type I collagen, osteopontin and bone sialoprotein [45]. In addition, the bone matrix is also rich in a variety of cytokines and growth factors [45]. The bone microenvironment contains many types of cells, such as osteoblasts, osteoclasts, haematopoietic stem cells, adipocytes and immune cells [46]. Under normal conditions, osteoblasts and osteoclasts establish a dynamic balance between bone formation and decomposition [46]. Bone metastasis often occurs in prostate cancer, breast cancer and lung cancer. Among these processes, bone metastasis of prostate cancer usually presents an osteoblastic metastasis phenotype, while breast cancer and lung cancer often present an osteolytic metastasis phenotype [46]. Metastatic cells interact with osteoblasts and osteoclasts in the bone microenvironment to promote the release of growth factors in the bone matrix and thus promote the growth of the cancer cells at the site of the metastasis, forming a “vicious cycle” of bone metastasis [46].

The metabolic adaptation of bone metastatic cells is mainly related to the specific metabolites, oxygen content and various cell types in the bone microenvironment. In mouse models of breast cancer, cancer cells are more likely to metastasize to sites containing immature HA, such as smaller, incomplete, and non-oriented crystallized HA sites [47]. Breast microcalcifications are common in the primary site of breast cancer. Therefore, radiography of breast microcalcifications is commonly used in clinical screening for breast cancer. Breast microcalcifications can be divided into two types. Type I microcalcifications are mainly composed of calcium oxalate crystals, which are common in benign tumours [48]. Type II microcalcifications are mainly composed of HA and are common in benign or malignant tumours [48]. Microcalcification in the primary site of breast cancer is related to cell proliferation and migration, inflammation and matrix degradation [48]. Breast cancer cells may increase the intracellular calcium ion concentration through phagocytosis and the degradation of intracellular crystals, thereby exerting cancer-promoting effects [49]. In addition, previous studies have shown that there is a pre-selection mechanism for cancer metastasis. Because of similarities between the primary microenvironment and the secondary microenvironment, the propensity for bone metastasis may pre-exist in the primary cancer [13]. The existence of the pre-selection mechanism means that the primary tumour microenvironment can be used to predict the organ specificity of cancer metastasis and provide markers for early clinical diagnosis and treatment. In view of the presence of HA in the microcalcification area and bone metastases in breast cancer, predictions on the tendency of bone metastasis based on the existence and morphological structure of microcalcification in the primary site remain to be explored. Prostate cancer, breast cancer, and renal cell carcinoma with a tendency to metastasize to bone can sense the extracellular Ca^2+^ concentration through calcium-sensitive receptors to promote cell proliferation and migration [50–52]. Therefore, whether metastatic cancer cells can increase the extracellular Ca^2+^ concentration through phagocytosis and degradation to exert biological effects remains to be determined.

In the primary site, hypoxia is an important feature of the tumour microenvironment [53]. Similarly, the bone metastatic microenvironment is also a hypoxic environment [44]. Hypoxia and HIF-1 expression can promote breast cancer bone metastasis and induce osteolytic metastasis by inhibiting osteoblast differentiation and promoting osteoclast production [54]. Moreover, ER-negative breast cancer cells with hypoxia at the primary site can secrete lysyl oxidase to promote the formation of premetastatic niches in osteolytic bone [55], revealing the close relationship between hypoxia and bone metastasis. Hypoxia in the primary site can lead to the production of a large amount of lactic acid, causing a reduction in extracellular pH [53]. The bone metastatic microenvironment can also induce the formation of an extracellular acidic environment due to the combined actions of osteoclasts and bone metastatic cancer cells [56]. Therefore, studying the similarity of metabolic microenvironments of the primary site and the metastatic site is helpful for exploring the mechanism of organotropism.

In addition, the specific cellular metabolic interactions in the bone metastatic microenvironment are also related to bone-specific metastasis. Serine metabolism is very important for osteoclastogenesis [57]. Compared with breast cancer cells with weak bone metastasis activity, the expression of de novo serine synthesis enzymes, including PHGDH, PSAT1, and PSPH, is increased in breast cancer cells with high bone metastasis activity [58]. Serine can stimulate the formation of human osteoclasts and promote their osteolytic activity [58]. Therefore, breast cancer cells may promote the formation of osteoclasts through the de novo synthesis of serine to promote a vicious cycle. The lactate released by MDA-MB-231 cells through glycolysis can be taken up by osteoclasts to provide energy, enhance their ability to degrade bone matrix, and promote the formation of osteolytic metastasis [59]. Osteogenic cells can deliver Ca^2+^ to cancer cells through gap junctions and activate downstream calcium signalling pathways and mTOR signalling pathways to support the progression of bone metastasis [60]. In addition to osteoclasts and osteogenic cells, the metabolic interactions between bone marrow adipocytes and cancer cells can also promote cancer cells adaptation to the bone microenvironment. Bone metastatic prostate cancer cells can take up lipids from bone marrow adipocytes through fatty acid-binding protein (FABP) 4 to promote cancer growth and invasion [61–63]. Bone marrow adipocytes can also promote the activation of HIF-1α in metastatic prostate cancer cells, resulting in metastatic cells acquisition of the Warburg effect phenotype [64]. In addition, primary cancer cells can also secrete exosomes to deliver pyruvate kinase M2 to bone marrow stromal cells (BMSCs) and upregulate CXCL12 of BMSCs in a HIF-1α-dependent manner, forming a premetastatic niche for bone metastasis of prostate cancer [65].

In summary, the current studies on the metabolic adaptation of bone metastasis mainly focus on the roles of inorganic components and bone-specific cells. However, most of the effects of bone inorganic components on cancer has been characterized based on in vitro tests, and more in vivo tests are needed to prove that these effects truly occur. The understanding of the effects of bone-specific cells on cancer metabolism is still limited to the interaction between cancer cells and other cells. Thus, more studies are needed to demonstrate how multiple cellular synergies affect the metabolism of bone metastases. Because osteolytic metastasis can degrade not only the inorganic component HA in the bone matrix but also the organic component, studies should be taken to determine whether osteolytic metastasis cells can directly obtain organic nutrients from the matrix by promoting osteoclast function.

### Liver

The liver is the metabolic centre, where liver cells complete most metabolic activities of the whole body to control substance and energy balances. The liver has a unique blood supply system composed of the hepatic artery and portal vein, which merge through the sinusoids and finally flow into the central vein [66]. Arterial blood from the hepatic artery is enriched with nutrients and oxygen, while venous blood from the portal vein contains food-derived substances such as lipid droplets and is deoxygenated [66]. Therefore, the liver has a hypoxic microenvironment. In addition, the liver microenvironment is composed of a variety of cells, including hepatocytes, hepatic stellate cells (HSCs), sinusoidal endothelial cells and many kinds of immune cells [67]. Liver metastasis often forms with breast cancer, melanoma and gastrointestinal cancer cells. The reason that the incidence of metastatic liver cancer is higher than that of primary liver cancer requires a two-part explanation. First, the special blood supply system of the liver and the high permeability of blood sinuses may provide appropriate secondary sites. Second, due to the natural tolerance of the liver to substances derived from intestinal microorganisms, an immune-tolerant environment is developed [68].

The metabolic adaptation of liver-specific metastatic cells is mainly related to competition for hepatic nutrients, the hypoxic environment, and the effects of intrahepatic cells. Metastatic liver cancer cells may compete with liver cells for nutrients needed for growth by acquiring metabolic capacity similar to that of health liver cells. Low-dose fructose is mainly metabolized by the small intestine, and high-dose fructose is mainly metabolized by colon microorganisms and the liver [69]. However, liver metastatic colon cancer cells can upregulate aldolase B via GATA6, thereby acquiring the ability to metabolize fructose and enhancing the proliferation ability of colon cancer cells [70]. A recent study showed that liver metastasis of colorectal cancer can induce epigenetic remodelling through enhancers or super enhancers, thereby causing the migrating colorectal cancer cells to lose their colorectum-specific transcription programme and acquire a liver-specific transcription programme [71]. In addition, this study also used the gene expression profiles of a large number of primary and metastatic cancers to show the universality of transcription characteristic elimination from primary cancer cells and transcription characteristics acquisition of secondary site cells [71]. More studies are needed to prove whether other types of metastatic cancer cells also acquire the transcriptional and metabolic characteristics of the metastatic site through epigenetic remodelling.

There is a hypoxic microenvironment in the liver that can easily lead to the depletion of ATP that causes metastatic cancer cells to die. Metastatic cancer cells in the liver from colorectal cancer can downregulate miR-483-5p and miR-551a to upregulate the expression of a brain-type creatine kinase and secrete it into the liver metastatic microenvironment, phosphorylating creatine to produce phosphocreatine [72]. Subsequently, phosphocreatine is taken up by the metastasized colorectal cancer cells to supply intracellular ATP, allowing them to survive under hypoxic conditions [72]. A study on the metabolic reprogramming of multiple organ metastases of breast cancer found that compared with breast cancer at the primary site, breast cancer cells that have metastasized to the liver, bone or lung show increased glycolysis and tricarboxylic acid cycle activity levels [73]. However, compared with that of bone and lung metastasis, the expression of HIF-1α and HIF-1α target PDK1 expression are at a higher level in liver cancer metastasis, resulting in stronger downstream glycolytic activity; however, oxidation phosphorylation and glutamine metabolism are weaker in the liver, which promotes the adaptation of cancer cells to the hypoxic environment in the liver [73]. Therefore, the hypoxic microenvironment in the liver forces metastatic cancer cells to undergo greater energy metabolism and enhances the characteristics of the Warburg effect.

Intrahepatic cells can also change the metabolic characteristics of liver metastases. Under normal physiological conditions, HSCs are in a resting state. However, upon triggered inflammation, HSCs are activated to become hepatic myofibroblasts (HMFs). The expression level of succinate dehydrogenase B (SDHB) is higher and the OXPHOS activity is stronger when pancreatic ductal epithelial cells (PDECs) were cocultured with HSCs than cocultured with HMFs. [74]. Knocking down SDHB can enhance cell proliferation and increase CSC characteristics [74]. Consistently, liver micrometastases (representative of a non-inflammatory environment) in KPC mice with pancreatic cancer exhibited higher expression of SDHB and lower expression of the CSC marker nestin than liver macrometastases (representative of a highly inflammatory environment) [74]. This finding shows that the activation level of HSCs and the inflammatory state of the liver can affect the metabolic state, proliferation ability and stem cell characteristics of PDECs. More evidence is needed to prove that other types of cells in the liver can affect the metabolic characteristics of liver metastases.

In summary, liver metastatic cells can acquire the specific metabolic ability of the liver and adapt to the hypoxic environment. The metabolic ability of liver metastatic cells can also be affected by other cells in the liver. In fact, there are other specific metabolic features of the liver, such as ketone body metabolism, enterohepatic circulation of bile acid, and ammonia metabolism, that may change the metabolic behaviour of cancer cells. For example, the aforementioned liver cancer cells can decompose ketone bodies to obtain substrates for energy metabolism [19]. Therefore, more studies are needed to demonstrate that other metabolic characteristics of liver cells can influence the metabolic behaviour of cancer cells that have metastasized to the liver.

### Lung

Lung metastasis often follows breast cancer, melanoma, and colon cancer [75]. The lung is the respiratory organ through which oxygen is inhaled and carbon dioxide is exhaled. Therefore, in contrast to the hypoxic environment of the bone and liver, the lung contains high oxygen levels, and cancer cells need to overcome the stress caused by oxidative damage to colonize in the lung. The abundant capillary network in the lung is conducive to cell adhesion and metastasis. However, the capillary endothelial cells are tightly connected, and therefore, cancer cells need to overcome the capillary barrier to colonize. There are many types of cells in the lung, including type I alveolar epithelial cells, type II alveolar epithelial cells, endothelial cells, fibroblasts, and various types of immune cells [75]. Furthermore, in addition to its respiration function, the lung also has many metabolic functions. While the lung plays a prominent role in lipid metabolism and the synthesis of pulmonary surfactant; on the other hand, it also synthesizes a variety of vasoactive substances, including amines, prostaglandins and peptides [76].

Currently, the metabolic adaptation of lung-specific metastasis mainly focuses on mitochondrial metabolism, antioxidant programmes, and the role of some metabolites or metabolic enzymes. Due to the oxygen-rich microenvironment in the lungs, metastasizing cancer cells can rewire their metabolism from glycolysis to OXPHOS. A recent study used a breast cancer PDX model and single cell transcriptome sequencing technology to prove that lung and lymph node micrometastases exhibit high levels of OXPHOS activity [77]. In contrast, primary breast cancer cells show upregulated glycolytic activity [77]. This conclusion was also verified by flow cytometry and metabolomics [77]. Mitochondrial complex V inhibitor oligomycin can be used to inhibit breast cancer OXPHOS and lung metastasis effectively but has no effect on primary breast cancer [77], indicating that OXPHOS has a profound targeting effect on lung metastasis, which also suggests that drugs targeting the mitochondrial electron transport chain can be used to target lung metastases. Peroxisome proliferation-activated receptor coactivator 1 alpha (PGC-1α) is a core regulator of mitochondrial energy production and oxidative metabolism, and its expression is often altered during cancer metastasis [78]. However, the specific mechanism of PGC-1α in cancer metastasis remains controversial. A study found that CTCs can increase the rate of mitochondrial OXPHOS, biosynthesis and oxygen consumption by upregulating PGC-1α to promote subsequent lung metastasis [79]. Silencing PGC-1α slows lung metastasis but does not affect primary cancer growth or the epithelial-mesenchymal transition (EMT) [79]. However, another study found no significant effect of the OXPHOS inhibitor metformin on metastasis in mice injected with breast cancer cells that highly express PGC-1α [80]. In contrast to the mechanism by which PGC-1α upregulates OXPHOS, this study indicated that PGC-1α enhances global energy metabolism flexibility of breast cancer cells, thereby producing resistance to bioenergy interference drugs such as metformin [80]. Therefore, the relationship between PGC-1α and cancer metastasis needs to be further studied.

Oxygen not only changes the energy metabolism of cancer cells but also exposes cancer cells to oxidative stress [81]. Therefore, antioxidant programmes are required in lung metastatic cancer cells. Peroxiredoxin-2 can promote breast cancer metastasis to the lung by regulating oxidative stress, and silencing peroxiredoxin-2 can inhibit breast cancer lung metastasis by inducing oxidative damage [82]. Flura-seq, a highly sensitive in situ sequencing technology, and mouse PDX breast cancer models were used in a study showing that compared with breast fat pad and brain micrometastases, lung micrometastases induce mitochondrial electron transport complex I, oxidative stress and antioxidation programmes [83]. This study confirmed an increase in lung metastasis-specific oxidative stress and the upregulation of antioxidant programmes in clinical samples [83]. In fact, at multiple stages of cancer metastasis, cancer cells must overcome oxidative stress while breaking away from the extracellular matrix, living in the circulatory system, and colonizing [79, 84, 85]. Therefore, the ability of cancer cells to adapt to oxidative stress may be acquired before they metastasize to the lung.

Many metabolites or metabolic enzymes can influence lung metastasis through different mechanisms. Pyruvate carboxylase (PC) catalyses pyruvate to produce oxaloacetate, which can then be used for gluconeogenesis or to replenish the TCA cycle. Lung metastatic breast cancer depends on PC. PC-deprived breast cancer cells show a decrease in glycolytic capacity and oxygen consumption rate and an increase in sensitivity to oxidative stress [86]. Through ^13^C tracing analysis, it was found that, compared with primary breast cancer, the concentration of mitochondrial pyruvate in lung metastatic breast cancer increases, thereby promoting PC-dependent replenishment through enzymatic kinetics [87]. Surprisingly, the growth of primary NSCLC also depends on PC [40, 88]. Therefore, factors in the lung microenvironment may enable both primary and metastatic cells to acquire a PC-dependent phenotype. However, the factors in the lung microenvironment that promote cancer cell acquisiton of a PC-dependent phenotype remain to be explored. Pyruvate can also be directly converted into α-ketoglutarate by alanine aminotransferase [89]. Subsequently, α-ketoglutarate activates P4HA through enzymatic kinetic metabolism, promotes collagen hydroxylation, extracellular matrix remodelling, and the growth of lung metastatic breast cancer [89]. Fatty acid metabolism can also affect ability of cancers to metastasize the lung. High expression of AKR1B10 in breast cancer cells show reduced glycolytic activity and dependence on glucose and enhanced fatty acid oxidation (FAO) [90]. In three-dimensional cancer spheroids formed in vitro and cancer metastases formed in vivo, FAO inhibitors blocked the metastasis of AKR1B10^High^ cancer cells but did not affect AKR1B10^Low^ cancer cells [90]. Further analysis confirmed that AKR1B10 can limit the side effects caused by of oxidative stress, thereby maintaining FAO in lung metastatic breast cancer [90]. Proline dehydrogenase catalyses the catabolism of proline. In a mouse model of lung metastasis, the expression of proline dehydrogenase in the lung metastatic cells was higher than primary cancer cells [91]. Inhibition of proline dehydrogenase effectively reduced the formation of lung metastasis in these mice [91]. Another study found that the bioavailability of asparagine affected the metastatic potential of breast cancer [92]. Knockdown of asparagine synthetase, L-asparaginase treatment or direct restriction of asparagine in the diet can reduce lung metastasis by breast cancer cells without affecting primary cancer growth [92]. Increasing dietary asparagine or enhancing the expression of asparagine synthetase can promote breast cancer metastasis to the lung [92]. Therefore, this study also reveals that the use of dietary therapy can be explored from the perspective of cancer metabolism to reduce the incidence of clinical cancer metastasis.

In summary, metastatic cancer cells in the lung can adjust their energy metabolism to aerobic oxidation and adapt to the high oxidative stress environment, and they can be affected by certain metabolites and metabolic enzymes. However, it is still unclear how the metabolic capacity of cells in the lung and the inherent metabolic functions of the lung affect the metabolism of lung metastases. In addition, because of the tight junctions of capillary endothelial cells in the lungs, metastatic breast cancer cells tend to show increased expression of prostaglandin-endoperoxide synthase 2 (PTGS2, also known as COX2) [93]. Prostaglandin, a product of PTGS2, can enhance capillary permeability. Cancer metabolism enhancement of lung capillary permeability to promote cell migration across the endothelium remains to be studied.

### Brain

The brain is protected by the blood–brain barrier and the blood-cerebrospinal fluid barrier forming a relatively isolated environment. Although these barriers can protect the brain from external harmful substances, they also block some therapeutic drugs from entering the brain [94]. Neurons are the functional units of the nervous system. According to the neurotransmitters released from their nerve endings, neurons can be classified into glutamatergic neurons and gamma-aminobutyric neurons. Therefore, cancer cells that can use these neurotransmitters for processes such as energy synthesis, biosynthesis, and antioxidative stress have tremendous survival and proliferation advantages. Brain metastasis often occurs following lung cancer, breast cancer or melanoma. According to the location of metastasis, secondary brain cancer be divided into brain parenchymal metastasis and leptomeningeal metastasis [94]. The latter is a cancer that has metastasized to the subarachnoid space. The metabolic microenvironments of the brain parenchyma and the subarachnoid space are completely different. The brain parenchyma is composed of many types of cells, including neurons, astrocytes, oligodendrocytes, which consume a large amount of oxygen and mainly procure a sufficient amount of fuel from glucose or ketone bodies produced in the liver to undergo oxidative metabolism [95]. Thus, the brain has the characteristics of low energy reserves, high energy supply and high energy consumption. The subarachnoid space is filled with cerebrospinal fluid, which is notably hypoxic and nutrient deficient [95]. Different metabolic microenvironments may also lead to different metabolic characteristics of brain metastases.

Currently, the metabolic adaptations necessary for brain-specific metastasis is mainly related to brain-specific metabolites and the interaction between brain cells and tumour cells. Proteomics analyses have revealed that breast cancer cells metastasized to the brain tend to enhance the expression of metabolic enzymes involved in glycolysis, TCA cycle, OXPHOS, pentose phosphate pathway and glutathione system. These findings indicate that brain metastatic breast cancer cells may, on the one hand, obtain energy through the oxidative metabolism of glucose and, on the other hand, respond to oxidative stress by enhancing the alternative glucose metabolism pathway and maintaining cell redox homeostasis, thus gaining an advantage in survival and growth in the brain microenvironment [96]. Comparing the gene expression profiles of brain metastases and extracerebral metastases of melanoma, the metastatic melanomas in the brain are enriched in the OXPHOS pathway [97]. By comparing OXPHOS gene expression, the metabolome and metabolic pathway activity in intracranial and subcutaneous xenograft melanoma mice, the conclusion that OXPHOS activity is increased in brain metastatic melanoma was also confirmed [97]. OXPHOS inhibitors can improve the survival rate of intracranial melanoma xenograft mice, resistant to MAPK inhibitors and inhibit brain metastasis in spontaneous brain metastasis of melanoma in mouse models [97]. Similarly, a study that infused ^13^C-labelled glucose during surgical resection of brain gliomas and brain metastases and then used ^13^C NMR spectroscopy to test related metabolites found that brain metastasis and primary brain tumour metabolism are very similar. These cells mainly used glucose for mitochondrial oxidation and for the production of lactic acid, alanine, glutamic acid, glutamine and glycine [98]. These findings show that glycolysis, the TCA cycle, OXPHOS and some nonessential amino acid synthesis pathway activity are enhanced in glioma and brain metastatic tumours. However, the study also found that less than 50% of the acetyl-CoA pool is derived from glucose in the blood, indicating that other substances supply the energy metabolism of these glioma and brain metastatic cells [98]. In addition to glucose metabolism, lipid metabolism has an impact on brain metastasis. The FABP family is involved in the uptake, transport and metabolism of fatty acids. High expression of FABP7 is associated with poor prognosis and a high incidence of brain metastasis in breast cancer patients [99]. Through in vivo and in vitro experiments, researchers discovered that FABP7 can promote HER2 + breast cancer cells adaptation to the brain microenvironment by supporting the glycolytic phenotype and lipid droplet storage [99]. Amino acid metabolism can also affect brain metastasis. Brain metastatic breast cancer cells can use gluconeogenesis and branched chain amino acid oxidation to promote the survival and growth of breast cancer in the brain microenvironment [100]. Acetate metabolism also promotes brain metastases. Both primary brain tumours and various types of brain metastatic cells can oxidize acetate to meet their energy requirements [43]. Therefore, the glucose metabolism, lipid metabolism, amino acid metabolism and acetic acid metabolism of brain metastatic tumours undergo different degrees of changes, indicating that brain metastatic tumours seem to have extraordinary metabolic flexibility, facilitating the adaptation of these cells to the complex and unique brain microenvironment.

The most common cell type in the brain is the neuron. Neurons and cancer cells can communicate through neurotransmitters to promote the colonization of brain metastases. Gamma-aminobutyrate (GABA) is a common neurotransmitter in the brain. Brain tumour samples obtained during neurosurgery for HER2 + and triple-negative breast cancer, revealed that brain metastasis of breast cancer has GABAergic characteristics; that is, the brain metastatic cells have high expression of GABA-related proteins, including GABA_A_ receptors, GABA transporters, GABA transaminase and glutamate decarboxylase [101]. Further in vitro studies found that brain metastatic breast cancer cells undergo GABA catabolism to increase NADH levels, which indicates metastatic breast cancer cells with proliferation advantages in the brain [101]. Glutamate is also a common neurotransmitter in the brain, and one of its receptors is the N-methyl-D-aspartate receptor (NMDAR). In glutaminergic synapses, glutamate released by presynaptic neurons is rapidly absorbed by postsynaptic neurons and astrocytes around synapses, which not only ensures the timeliness of synaptic conduction but also prevents the toxic effect of high extracellular concentrations of glutamate on nerve cells [102]. A recent study found that breast cancer cells can replace astrocytes and form pseudo-tripartite synapses with glutamatergic neuron cells [103]. Metastatic cells can use glutamate released by neuronal cells to activate NMDAR receptors, thereby promoting brain metastasis [103]. In addition to neurons, other cells in the brain can also metabolically interact with brain metastatic cells through different mechanisms. Astrocytes can sequester calcium ions in the cytoplasm of melanoma cells through gap junctions and promote the chemotherapy resistance of melanoma cells in brain metastases [104]. Brain metastatic tumour cells can also deliver the second messenger cGAMP to astrocytes through gap junctions, activate the STING signalling pathway in astrocytes, and produce the inflammatory factors IFNα and TNF. Subsequently, inflammatory factors activate the STAT1 and NF-κB signalling pathways of brain metastatic cells through paracrine signalling to promote tumour cell growth and drug resistance [105]. Therefore, brain metastatic cells can promote the process of colonization through metabolic interactions with brain cells.

In summary, brain metastatic cells have a high degree of metabolic reprogramming, and they can also interact with neurons and astrocytes through neurotransmitters and gap junctions. However, the metabolic interactions between other types of cells and brain metastatic cells remain to be studied, and the differences in the metabolism of parenchymal brain metastasis and leptomeningeal metastasis also needs to be studied. In addition, similar to breast cancer cells with a tendency for lung metastasis, the expression of PTGS2 in breast cancer cells with a tendency for brain metastasis is also increased [106], suggesting that lung and brain metastatic breast cancer may pass through tight capillary connections through similar tumour metabolic mechanisms. In fact, a study found that PTGS2 is involved in the formation of cerebrospinal fluid tumour cells in brain metastatic breast cancer, which also indicates that PTGS2 is closely related to brain metastasis [107].

### Others

Approximately 80% of serous ovarian cancer metastasizes to the omentum. The omentum is rich in adipocytes and stores a large amount of lipids, which can promote omentum-specific metastasis of ovarian cancer [108]. Through the coculture model of adipocytes and ovarian cancer cells, adipocytes were found to transport lipids to ovarian cancer cells and promote cancer cell growth in vivo and in vitro [109]. Further studies revealed that the coculture model can induce lipid decomposition of adipocytes, promote β-oxidation of cancer cells, and enhance the acquisition of rich sources of energy by cancer cells [109]. In a comparison of primary ovarian cancer and omental metastasis, FABP4 was found to be upregulated in omental metastatic ovarian cancer and is located at the interface between adipocytes and cancer cells [109]. FABP4-deficient mice with ovarian cancer have reduced omental metastasis, confirming that adipocyte-derived lipids play important roles in the omental metastasis of ovarian cancer [109]. Another study found that in a coculture model consisting of adipocytes and ovarian cancer cells, the expression of the fatty acid receptor CD36 on the membrane of the ovarian cancer cells is increased, promoting the uptake of fatty acids by ovarian cancer cells [110]. In addition, CD36 plays a role in multiple processes of ovarian cancer metastasis to the omentum, including adhesion, invasion, migration and non-anchored growth [110]. Therefore, inhibition of CD36 can effectively inhibit ovarian cancer metastasis. Secreted protein acidic and rich in cysteine (SPARC) can inhibit omental metastasis of ovarian cancer by inhibiting the interaction between ovarian cancer cells and adipocytes, thereby inhibiting the phenotypic plasticity of omental adipocytes and metabolic reprogramming [111]. Among the inhibitory mechanisms, the effect of SPARC on metabolism is mainly to inhibit the metabolic reprogramming of adipocytes and ovarian cancer cells, inhibit adipocyte differentiation, and inhibit adipocytes from acquiring cancer-related phenotypes [111]. Compared with its level in primary ovarian cancer, salt-induced kinase 2 (SIK2) is highly expressed in lipid-rich metastases [112]. Adipocytes can activate the phosphorylation and activation of SIK2 in ovarian cancer cells in a calcium-dependent manner. On the one hand, activated SIK2 can activate the PI3K-AKT signalling pathway to promote cancer cell proliferation and survival; on the other hand, it can inhibit acetyl-coenzyme A carboxylase in combination with AMPK phosphorylation, increasing the expression of carnitine palmitoyl transferase 1 and activating FAO, thereby promoting omental metastasis of ovarian cancer [112]. In addition to lipid metabolism, omental adipose stromal cells can induce increased nitric oxide synthesis in ovarian and endometrial cancer cells, thereby promoting cancer cell proliferation [113]. Nitric oxide is catalysed from arginine via nitric oxide synthase, and citrulline is also produced. Research using a model of cocultured omental adipose stromal cells and cancer cells found that the cancer cells absorb arginine secreted by the omental adipose stromal cells and then secrete citrulline into the microenvironment [113]. Citrulline can increase adipogenesis in omental adipose stromal cells [113]. Further studies found that the increased nitric oxide synthesis in cancer cells induced by omental adipose stromal cells can upregulate glycolysis, reduce mitochondrial respiration, and reduce oxidative stress [113]. In addition to adipocytes, cancer-associated fibroblasts (CAFs) can also affect omental metastasis of ovarian cancer through cancer metabolism. With a model of cocultured omentum-derived CAFs and ovarian cancer cells, it was found that the activation of p38α MAPK in the CAFs promote the phosphorylation and activation of the glycogen metabolism enzyme glucose phosphate mutase 1 under normoxic conditions in the cancer cells [114]. Subsequently, the glycogenolysis of the cancer cells is increased, which supplies glycolysis and promotes cancer cell proliferation, invasion and metastasis [114]. Further studies using in vivo models showed that the depletion of p38α in CAFs and the inhibition of glycogenolysis in cancer cells can reduce omental metastasis [114]. Therefore, the propensity for omentum metastasis is related to multiple cell types and multiple metabolic pathways, which is of great significance to our understanding of the propensity for cancer metastasis.

Lymph node metastasis is often used to predict the risk of distant metastasis and death. It is also closely related to the prognosis of clinical patients and the choice of treatment method. A number of studies have shown that lymph node metastasis can be used as a foothold for distant metastasis [115, 116]. However, some studies have shown that lymph node metastasis is not the only way for distant metastasis [117]. Metabolic reprogramming studies of lymph node metastatic tumours mainly focus on lipid metabolism. In melanoma and breast cancer cells metastasized to lymph nodes, accumulated bile acid activates YAP through the vitamin D receptor, which leads to FAO activation of the cancer cells and facilities their adaptation to the lymph node microenvironment [118]. The long-chain noncoding RNA LNMICC recruits nuclear factor NPM1 to the promoter of FABP5 to reprogramme fatty acid metabolism and then promotes cervical cancer cell metastasis to lymph nodes through the EMT and VEGF-C-mediated lymphangiogenesis [119]. Therefore, lipid metabolism-targeted drugs may inhibit lymph node metastasis, thereby delaying cancer progression. In fact, the use of the FAO inhibitor etomoxir, a hypoglycemic agent, can effectively inhibit lymph node metastasis [118].

## Conclusions

### Driving effects of the metabolic microenvironment on cancer metastasis

**Fig. 2:**
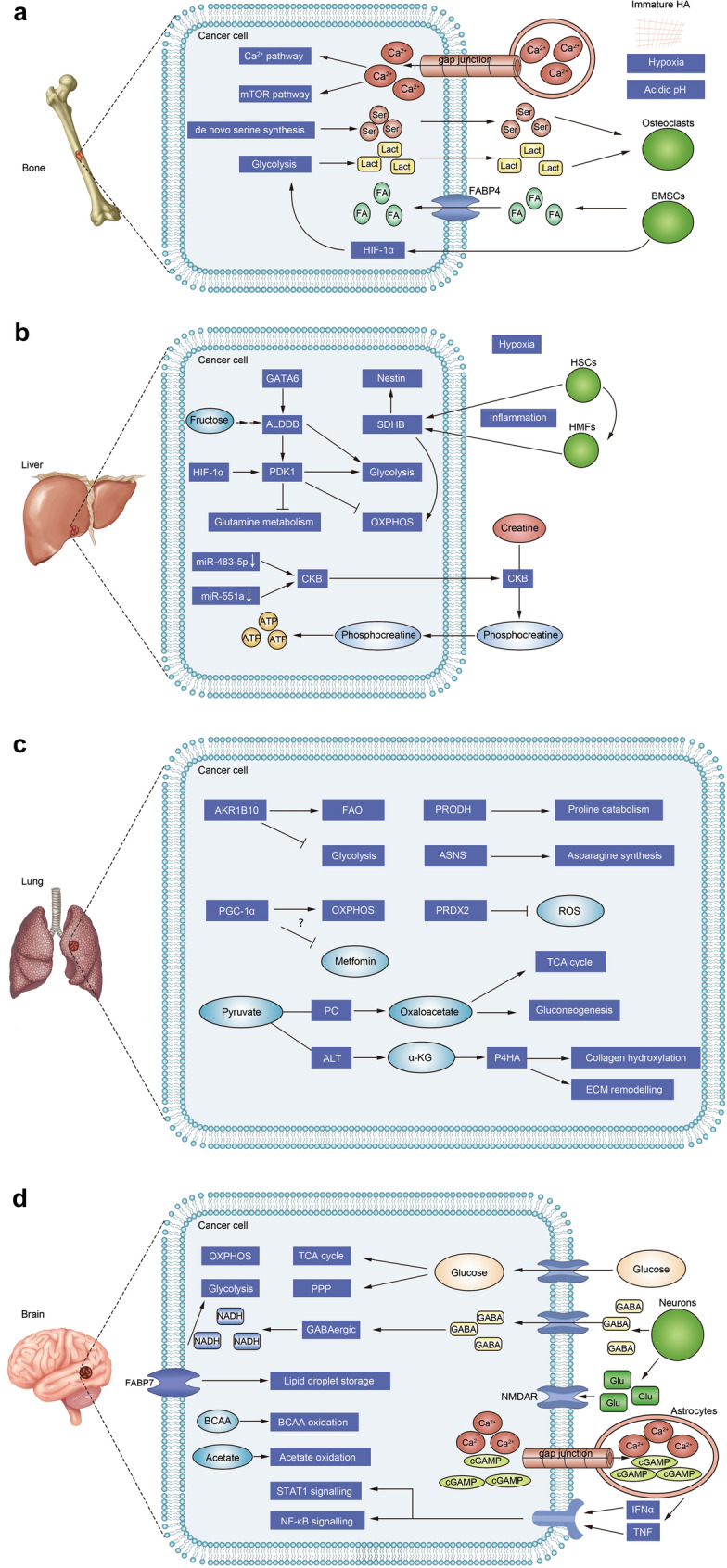
Metabolic adaptation mechanism of metastatic organotropism. Cancers that metastasize to bone (**a**), liver (**b**), lung (**c**) and brain (**d**), omentum, and lymph nodes need to adapt to different metabolic microenvironments to survive and grow at a distant site. Metastasizing cancer cells need to adapt to the metabolic microenvironments of the secondary organ, which mainly includes changes in energy and nutrient sources, organ-specific metabolites, pH, the degree of hypoxia, and the metabolic interactions between organ-specific cells and cancer cells. *HA* hydroxyapatite, *Ser* serine, *Lact* lactate, *FA* fatty acid, *BMSCs* bone marrow stromal cells, *ALDOB* aldolase B, *HSCs* hepatic stellate cells; *HMFs* hepatic myofibroblasts, *OXPHOS* oxidative phosphorylation, *CKB* brain-type creatine kinase, *FAO* fatty acid oxidation, *PGC-1α* peroxisome proliferation-activated receptor co-activator 1 alpha; *PRDX2* peroxiredoxin-2, *PRODH* proline dehydrogenase, *ASNS* asparagine synthetase, *PC* pyruvate carboxylase, *ALT* alanine aminotransferase, *α-KG* α-ketoglutarate, *ECM* extracellular matrix, *GABA* gamma-aminobutyrate, *PPP* pentose phosphate pathway, *Glu* glutamate, *BCAA* branched chain amino acid

Increasing evidence shows that the metabolic microenvironment plays important roles in driving metastasis. On the one hand, as mentioned above, cancer cells need to adapt to the metabolic microenvironment of the secondary environment to survive and proliferate (Fig. 2). On the other hand, cancer cells that cannot adapt to the primary metabolic microenvironment may leave the original site and colonize a microenvironment that better suited to their metabolic needs. Hypoxic lung cancer cells leave the oxygen-rich lung microenvironment and metastasize to hypoxic environments [120]. Liver cancer cells with vigorous mitochondrial metabolism can leave the hypoxic liver microenvironment and metastasize to the oxygen-rich lung microenvironment [121]. Therefore, studying the specific metabolic characteristics of various tissues and organs will help us further understand the reasons for cancer metastasis to secondary sites. In addition, designing drugs based on the metabolic characteristics of various tissues and organs will enable the targeting of metastasis from a new perspective.

### Process of mutual adaptation between seeds and the soil

While paying attention to the different metabolic microenvironments of different metastatic organs, we need to also pay attention to the dynamic changes of the metabolic microenvironment of the metastatic organ. On the one hand, primary tumours can transform the remote metabolic microenvironment of secondary organs through different mechanisms, which is the process of forming premetastatic niches. On the other hand, as the cancer reaches the secondary organs and changes from a micrometastasis to a macrometastasis, the microenvironment of the secondary organs is constantly modified. Therefore, the metabolic adaptation process of organ-specific metastasis is not merely a process in which the cancer cells constantly change; it is also a process in which cancer cells and metastatic organs continuously adapt, influence and change each other.

### Selection or adaptation?

Is the metabolic adaptation mechanism of metastatic cells the result of selection or adaptation? If it is the result of selection, then the driving factors of the metabolic phenotype likely originate from mutations, copy number variations and other genomic changes. Therefore, we may be able to identify the driving genes critical for metabolic phenotype changes in metastatic cancer cells through large-scale metastatic cancer genome resequencing. If the metabolic adaptation mechanism is the result of adaptation, than the factors driving the acquisition of the metabolic phenotype are reversible mechanisms, and are derived from the environment, likely in relation to epigenetic mechanisms. Therefore, in the process of organ-specific metastasis, changes in the environment may induce epigenetic changes, leading to changes in metabolism. Metabolism can also affect epigenetic modifications [17], thereby further promoting organ-specific metastasis. Because epigenetic changes are fast and reversible compared to mutations, they may be more flexible and common.

### Treating CTCs as a form of circulatory system metastasis

When studying the metabolic adaptation mechanism of metastasis, we can regard CTCs as a form of circulatory system metastasis and explore how cancer cells match the metabolic microenvironment of the circulatory system. After non-transformed cells detach from the extracellular matrix of the primary site, they often undergo increased intracellular ROS and anoikis. Cancer cells can resist this process by enhancing the intracellular antioxidant programme [122]. Therefore, the current research with metabolic models of CTCs mainly focuses on antioxidant metabolism. However, treating CTCs from the perspective of metabolic adaptation, we may discover more possible research directions, such as the influence of the difference in arterial and venous oxygen or nutrient content in the circulatory system, and the influence of metabolic interactions between various cells in the circulatory system and cancer cells.

### Application of emerging technologies

The application of emerging technologies can produce more in-depth insights into the metabolic adaptation mechanism of organ-specific metastasis. Single-cell transcriptome sequencing combined with PDX models can be used to study rare cell subpopulations during cancer metastasis. For example, studies on the micrometastatic clones formed by DTCs may reveal a more refined metastatic process [77]. Using in situ transcriptome sequencing technology combined with a PDX model not only enables study into the changes in the expression of metabolic enzymes but also the spatial location information of metastatic cells [83] to gain understanding into spatially specific metabolic reprogramming during metastasis. Moreover, this technology is helpful for studying the metabolic differences of cancer cells in different environments within the same primary or metastatic tumour. Metabolomics and metabolic flux analysis, the two major metabolic high-throughput analysis methods, may significantly enhance the overall capabilities for comprehensive research in this field. The combination of transcriptomics, proteomics, metabolomics and other multi-omics technologies will also reveal the mechanisms of cancer metastasis and metabolic reprogramming on multiple levels.

### Clinical significance of metastatic adaptation mechanisms

According to the metabolic adaptation mechanism of cancer metastasis, different cancer cells that metastasize to the same organ have similar metabolic characteristics. Therefore, designing therapeutic targets for these metabolic characteristics may provide new ideas for the prevention and treatment of cancer metastasis. Since liver metastasis is usually more active in anaerobic glycolysis, we can try to use respiratory chain inhibitors to prevent the process of lung metastasis. By contrast, we can also try to use glycolysis inhibitors to interfere liver metastasis which has an active anaerobic glycolysis mode. In addition to targeting the metabolic process of cancer cells, it is also possible to try to change the inherent metabolic characteristics of certain organs with a high frequency of metastasis, making it difficult for cancer cells to adapt to corresponding organs. The bone matrix degradation within the metastatic microenvironment plays a vital role in osteolytic metastatic cancer. Therefore, inhibiting this process may hinder bone metastasis. The local acidic environment of liver and bone may have a certain impact on the adaptability of metastatic cells. As a result, adjusting the pH of local organs through oral administration or local addition has great potential for the treatment of metastasis. In fact, existing studies have found that bicarbonate can markedly enhance the anticancer activity of transarterial chemoembolization [123]. In the terminal stage of clinical cancer patients, cancer cells mostly pass blood or lymphatic vessels to reach multiple organs throughout the body. Targeting circulatory or lymphatic metastasis may be able to prevent or inhibit this deadly process. Hence, we can try to use hemofiltration or other devices to adjust the concentration of some metastasis-specific nutrients in these vessels, thereby inhibiting the process of multiorgan invasion.

## Data Availability

Not applicable.

## Abbreviations

CTCs: Circulating tumour cells
DTCs: Disseminated tumour cells
OXPHOS: Oxidative phosphorylation
CP: Carbamoyl phosphate
NSCLC: Non-small cell lung cancer
CPS: Carbamoyl phosphate synthase
UMPS: Uridine monophosphate synthase
HA: Hydroxyapatite
FABP: Fatty acid-binding protein
BMSCs: Bone marrow stromal cells
HSCs: Hepatic stellate cells
HMFs: Hepatic myofibroblasts
SDHB: Succinate dehydrogenase B
PDECs: Pancreatic ductal epithelial cells
PGC-1α: Peroxisome proliferation-activated receptor co-activator 1 alpha
EMT: Epithelial-mesenchymal transition
PC: Pyruvate carboxylase
FAO: Fatty acid oxidation
PTGS2: Prostaglandin-endoperoxide synthase 2
GABA: Gamma-aminobutyrate
NMDAR: N-methyl-D aspartate receptor
SPARC: Secreted protein acidic and rich in cysteine
SIK2: Salt-induced kinase 2
and CAFs: Cancer-associated fibroblasts
